# Molecular Carrier-Assisted Self-Assembly of *st*-PMMA Helical Complexes for Fluorescence Sensing of Nitroaromatics in Aqueous Medium

**DOI:** 10.1021/acsami.5c13301

**Published:** 2025-09-11

**Authors:** Yu-Hao Wang, Wen-Tsung Tseng, Kuan-Yi Wu

**Affiliations:** Department of Chemical Engineering and Biotechnology, 34877 National Taipei University of Technology, Taipei 10608, Taiwan

**Keywords:** molecular recognition, cooperative self-assembly, inclusion complex, stereoregular polymer, nitro-aromatic compounds

## Abstract

In nature, the cooperative binding of enzyme, coenzyme, and substrate forms a functional ternary complex that activates catalytic activity essential for biological functions. Inspired by this mechanism, we present a supramolecular strategy employing pyrene as a molecular carrier to facilitate the encapsulation of nonbinding nitroaromatic compounds (NACs) into the helical structure of syndiotactic poly­(methyl methacrylate) (*st*-PMMA) via charge-transfer (CT) interactions. Structure characterization revealed that NACs and *st*-PMMA only form amorphous mixtures due to the weak binding affinity. In contrast, pyrene guests can coassemble with *st*-PMMA host to form helical complexes. Upon adding NACs to the *st*-PMMA/pyrene system, CT complexation between pyrene and NACs occurs readily in solution. Given the specific molecular recognition between pyrene and the *st*-PMMA host, pyrene can act as the carrier, enabling the pyrene-tagged NACs to be subsequently encapsulated within the *st*-PMMA helix during the transition to the solid state. Spectroscopic analyses confirm that the nonradiative CT complex of pyrene/NACs suppresses pyrene excimer formation in the *st*-PMMA helix and leads to the fluorescence quenching. Notably, owing to the dynamic nature of molecular carrier-induced self-assembly, the transparent *st*-PMMA/pyrene complex films demonstrate effective detection of explosive NACs in aqueous medium. Therefore, these findings establish a cooperative self-assembled strategy for modulating inclusion behavior in helical polymers, offering new avenues for advanced sensing platforms and precision-controlled molecular encapsulation.

## Graphical abstract

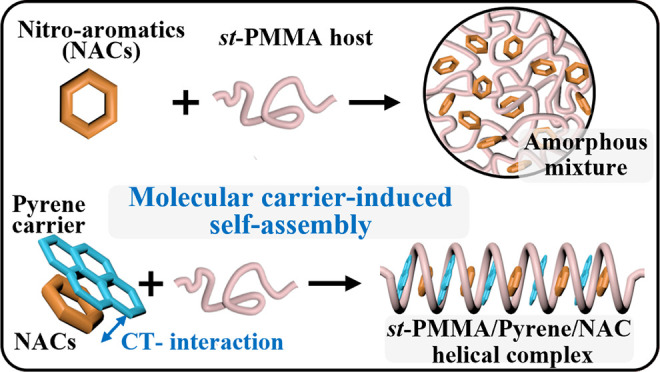

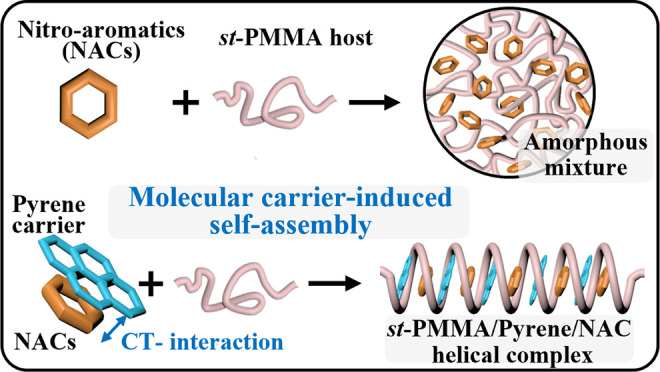

## Introduction

The essential biological functions of living organisms arise from molecular recognition processes within fundamental biomolecular assemblies such as enzymes, DNA, and lipid membranes.^1,2^ These recognition events can be effectively interpreted through the framework of host–guest chemistry, wherein host molecules, such as protein enzymes or biological receptors, selectively bind complementary substrate guests.^3,4^ Upon molecular recognition, the formation of host–guest complex often undergoes conformational changes through an induced-fit mechanism, thereby triggering specific functional responses.^5–7^ However, many protein enzymes possess incomplete structural configurations or insufficient intrinsic affinity to stably bind substrates on their own.^8^ To overcome this limitation, biological systems employ nonprotein molecules, known as coenzymes, which initially associate with the enzyme to form an enzyme–coenzyme complex.^9^ This coassembly modulates the conformation of protein binding pockets and enhances the binding affinity toward substrate molecules. The resulting cooperativity of protein and coenzyme facilitates the subsequent incorporation of substrate molecules to form a ternary enzyme–coenzyme–substrate complex capable of efficient catalysis.^10^ Thus, nature employs cooperative self-assembly as a refined strategy to achieve functional complexity in biological systems.^11^

In host–guest chemistry, helical polymers that possess a unique confined nanospace can act as the host for encapsulating specific guests.^12–14^ This unique behavior of helical polymers has been attracting significant attention in the fields of sensing, separation, catalysis, nanoreactor, and electronics.^14–16^ Among the synthetic helical polymers, syndiotactic poly­(methyl methacrylate) (*st*-PMMA) is the representative example that shows the helical wrapping behavior similar to DNA strands.^14^ *st*-PMMA hosts can conduct the helical self-assembly with specific π-conjugated compounds like phenanthrene, pyrene, C_60_, etc., through molecular recognition.^17–19^ Following the induced-fit mechanism, the coiled *st*-PMMA wraps the guest molecules to form the supramolecular helix. The nanocylindrical space with a diameter of ca. 1 nm makes the aromatic guests align into a 1-dimensional array. The nanoscale confinement modulates the spatial packing and electronic interactions among the π-conjugated guests, giving rise to emergent physicochemical properties that differ significantly from those observed in their bulk or aggregated states.^13,20–22^

In the *st*-PMMA multicomponent systems, it is well-known that *st*-PMMA hosts exhibit selective self-assembly with various guest molecules, primarily due to the absence of the cooperative guest–guest interactions.^14,23^ Therefore, the competitive encapsulation of distinct guests is merely dependent on the differences in binding affinity to the *st*-PMMA helical host, making *st*-PMMA hosts with promising potential for molecular separation.^24^ For instance, *st*-PMMA has demonstrated size-selective encapsulation, favoring the inclusion of larger fullerenes (e.g., C_76_ and above), thus enabling efficient separation strategies based on molecular size discrimination.^25,26^ However, it remains unclear whether the involvement of favorable noncovalent interactions between distinct guest species, such as π–π stacking or charge-transfer (CT) interactions, can induce cooperative self-assembly within the *st*-PMMA helix.^27,28^ Addressing this issue would unlock cooperative self-assembly in the *st*-PMMA supramolecular systems and bring out novel functions in addition to the self-sorting behavior from selective self-assembly.

Inspired by the cooperativity in biological systems, this study develops a molecular carrier-assisted self-assembly strategy in which the pyrene guest acts as the molecular carrier, facilitating the encapsulation of nonbinding molecules into the *st*-PMMA helices. As shown in Figure 1, pyrene guests can coassemble with the *st*-PMMA host to form a helical inclusion complex (HIC). In contrast, electron-deficient nitroaromatic compounds (NACs) such as 4-nitrotoluene (4-NT), 2,4-dinitrotoluene (2,4-DNT), and 2,6-dinitrotoluene (2,6-DNT) exhibit negligible binding affinity toward *st*-PMMA, resulting only in the amorphous mixture. However, upon introducing NACs into the *st*-PMMA/pyrene system, NACs are effectively tagged by pyrene carriers through the CT interaction, enabling the formation of CT complexes in the *st*-PMMA solution. During solidification, these pyrene/NACs CT complexes can be recognized by the *st*-PMMA hosts and subsequently wrapped into the helical cavity. Structural and optical analyses confirm that the nonradiative CT complex disrupts pyrene excimer formation and leads to pronounced fluorescence quenching. This fluorescence quenching demonstrates potential for detecting explosive NACs in aqueous media. As a result, this work presents a novel supramolecular design strategy to achieve molecular encapsulation via carrier-mediated inclusion, paving the way for stimuli-responsive nanomaterials.

**Figure 1:**
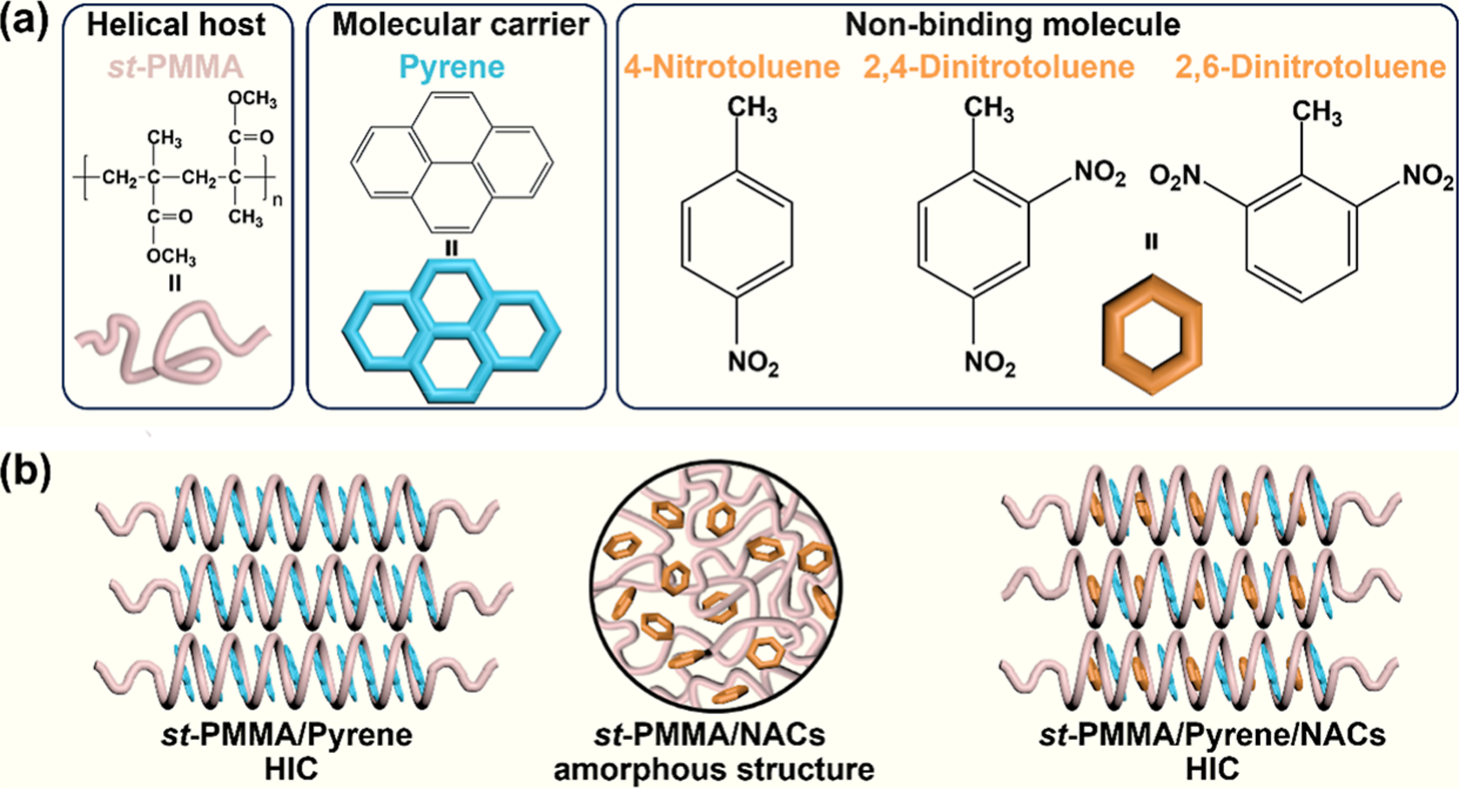
(a) Chemical structures of *st*-PMMA, pyrene, and nitro-aromatic compounds (NACs). (b) Illustration of supramolecular structures of the *st*-PMMA/pyrene, *st*-PMMA/NACs, and *st*-PMMA/pyrene/NACs systems. (Note: The methyl and ester methyl side groups of *st*-PMMA are omitted for clarity.).

## Results and Discussion

### Self-Assembled Behavior of the *st*-PMMA/Pyrene Complex system

*st*-PMMA with a *M*_w_ of 35 kg/mol and a *rr* content of ca. 86% was chosen in this study. The molecular characterization of the *st*-PMMA by the ^1^H nuclear magnetic resonance (NMR) and gel permeation chromatography is shown in Figures S1 and S2. Besides, the detailed procedure for preparing the *st*-PMMA/pyrene blends with varying pyrene contents (0–22 wt %) is also depicted in the Experimental Section. Figure 2a first presents optical microscopy (OM) images for comparing the morphologies of different samples. As gradually increasing [pyrene] to 20 wt %, the *st*-PMMA/pyrene mixture appears as a colorless and transparent film, markedly different from the diamond-shaped microcrystals of pyrene (Figure 2a). Moreover, the corresponding fluorescence (FL) images (Figure 2b) display that the emitted light-blue color results from the pyrenes. The intensity gradually increases with the pyrene content in the *st*-PMMA/pyrene transparent film. The FL spectrum in Figure 2b further confirms that the dominant FL emission at λ_em_ = 475 nm can be attributed to the formation of pyrene excimers in the *st*-PMMA matrix instead of pyrene monomers.^29^ These morphological features in the *st*-PMMA/pyrene mixture show that pyrene excimers are homogeneously distributed within the *st*-PMMA matrix at [pyrene] up to 20 wt % without pyrene crystallization.

**Figure 2:**
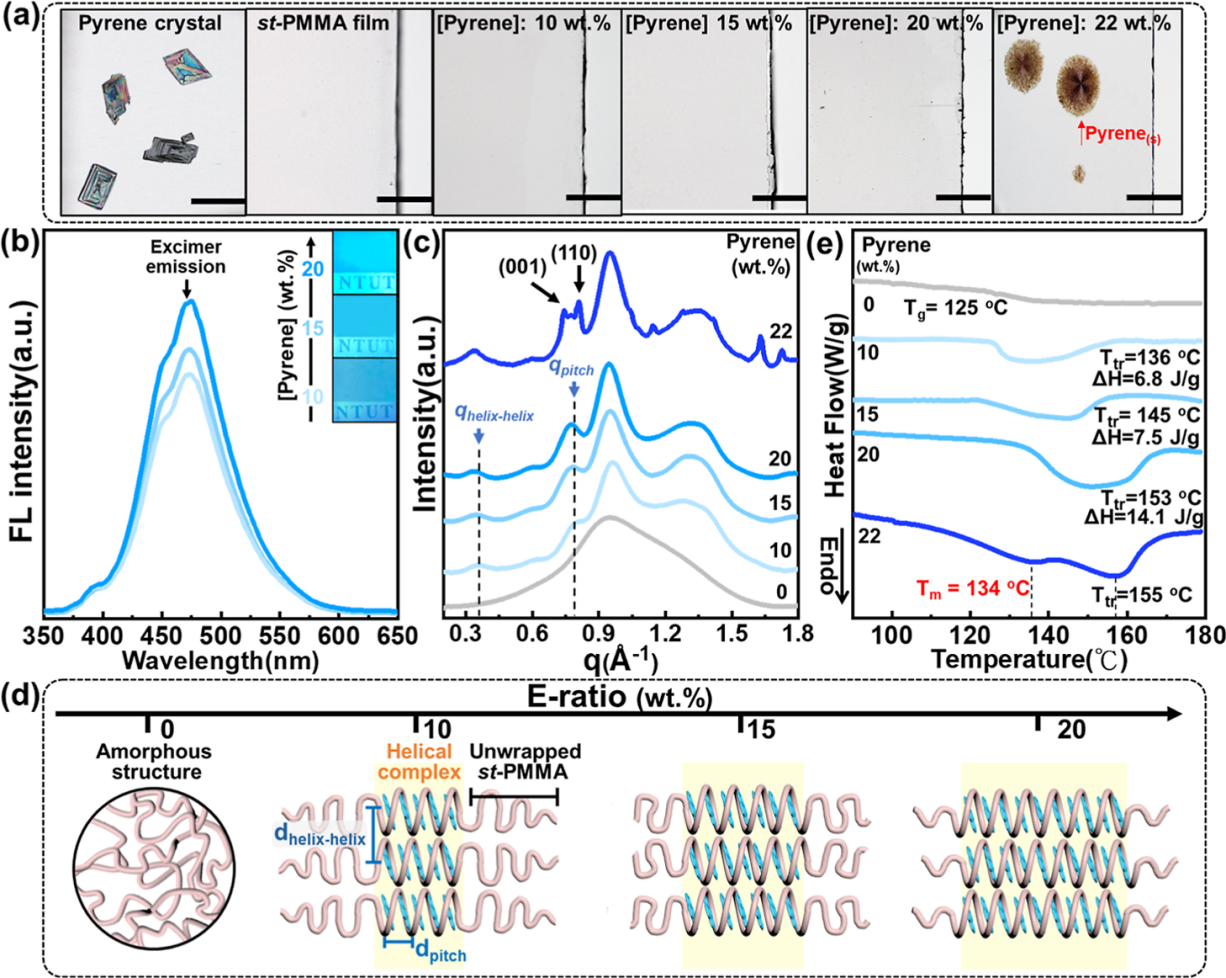
Structural characterization of *st*-PMMA/pyrene complex morphology: (a) OM micrographs of pyrene crystals, *st*-PMMA amorphous film, and the *st*-PMMA/pyrene complex film with varying pyrene contents (0 wt % to 22 wt %). Scale bar: 100 μm. (b) Corresponding FL spectrum, excited at 340 nm. Insets are the photographs excited by a UV lamp (λ_ex_ = 254 nm). (c) WAXD profiles of *st*-PMMA/pyrene complex film with different pyrene contents. (d) E-ratio-dependent hierarchical structure in the *st*-PMMA/pyrene complex film. (e) DSC thermograms of *st*-PMMA/pyrene complex films with varying pyrene contents. (Note: the methyl and ester methyl side groups of *st*-PMMA in Figure 2d are omitted for clarity).

Wide-angle X-ray diffraction analysis (WAXD) is further utilized to disclose the microscopic structure in the *st*-PMMA/pyrene homogeneous and transparent morphology. WAXD profiles in Figure 2c confirm the amorphous feature of neat *st*-PMMA film due to the broad scattering hump between *q* = 0.4 and 1.6 Å^–1^. Upon adding [pyrene] to 10 wt %, the diffraction pattern exhibits semicrystalline characteristics, featuring a broad scattering signal associated with the amorphous *st*-PMMA structure, along with several distinct diffraction peaks corresponding to the *st*-PMMA/pyrene HICs. Specifically, the peaks at *q*_helix–helix_ = 0.356 Å^–1^ and *q*_pitch_ = 0.782 Å^–1^ are attributed to the interhelix distance (*d*_helix–helix_ = 17.6 Å) and the helical pitch (*d*_pitch_ = 8.03 Å) of the HIC, where a 1-dimensional molecular array of pyrenes is confined in the *st*-PMMA supramolecular helix, illustrated in Figure 2c,d.^14,17^ As further increasing [pyrene] to 20 wt %, the diffraction intensity becomes higher and sharper, indicating the increase in the composition of *st*-PMMA/pyrene HIC in the semicrystalline morphology. Furthermore, Figures 2c and S3 display that at 22 wt %, several sharp diffraction peaks of pyrene crystals appear in the *st*-PMMA/pyrene mixture. This result indicates that the highest encapsulation ratio (E-ratio) of pyrene guests in the *st*-PMMA helical host is approximately 20 wt %, consistent with the OM observation (Figure 2a).

Thermal analysis of the *st*-PMMA/pyrene complex is also studied to understand the thermostability of the *st*-PMMA/pyrene complex as a function of its composition. Figure 2e reveals the differential scanning calorimetry (DSC) thermograms for *st*-PMMA/pyrene complexes with various pyrene E-ratios ranging from 0 wt % to 20 wt %. For the *st*-PMMA amorphous structure, the thermogram displays only the glass transition temperature (*T*_g_) at 125 °C. At the E-ratio of 10 wt %, an endothermic peak (Δ*H* = 6.8 J/g) appears at 136 °C, corresponding to the phase-transition temperature (*T*_tr_) of the *st*-PMMA/pyrene complex, where *st*-PMMA hosts start to unwrap the pyrene guests. Upon increasing the E-ratio to 20 wt %, *T*_tr_ further shifts from 136 to 153 °C, accompanied by a rise in enthalpy change (Δ*H*) to 14.1 J/g. These results reconfirm that a higher E-ratio not only enhances the formation of larger grains of the *st*-PMMA/pyrene helical complex but also promotes the composition of *st*-PMMA/pyrene HICs within the semicrystalline morphology, as illustrated in Figure 2c,d. Compared to previous studies involving *st*-PMMA with nearly perfect tacticity (*rr* > 94%), the *st*-PMMA/pyrene HICs prepared with a lower *rr* value of 86% exhibit a reduction in T_tr_.^18^ This decline is likely attributed to higher stereoirregularity in the *st*-PMMA (*rr* = 86%) backbone, which may disrupt regular helix formation and thus reduce the structural order of the HICs. Moreover, adding [pyrene] = 22 wt %, the additional *T*_m_ appears at 134 °C, corresponding to the melting of pyrene crystals. This result reconfirms that the highest E-ratio of pyrene in the *st*-PMMA helix (*rr* = 86%) is approximately 20 wt %.

### Structural Characterization of the *st*-PMMA/NACs Blend system

Previous studies have established that *st*-PMMA can form HICs with electron-rich aromatic compounds such as toluene, *m*-xylene, pyrene and phenanthrene.^30,31^ However, the capability for *st*-PMMA to accommodate electron-deficient NACs, including 4-NT, 2,4-DNT, and 2,6-DNT, has yet to be investigated. As shown in Figure S4, OM images reveal that the *st*-PMMA/NACs blend films remain transparent and colorless even with increasing NAC content loading up to 20 wt %, suggesting good miscibility between *st*-PMMA and NACs in the solid state. Nevertheless, composition-dependent WAXD profiles (Figure 3a–c) show only a broad amorphous halo with the increasing intensity centered at *q* = 1.2 Å^–1^ as the NACs contents increase. Notably, no diffraction features associated with the formation of the helical complex are detected, indicating the absence of ordered inclusion structures. Unlike pyrene guests (Figure 2), these NAC molecules exhibit insufficient binding affinity toward the *st*-PMMA helix, thereby failing to induce the supramolecular wrapping.^26^ As a consequence, the *st*-PMMA/NACs system remains amorphous without forming the HIC structures, as illustrated in Figure 3d.

**Figure 3:**
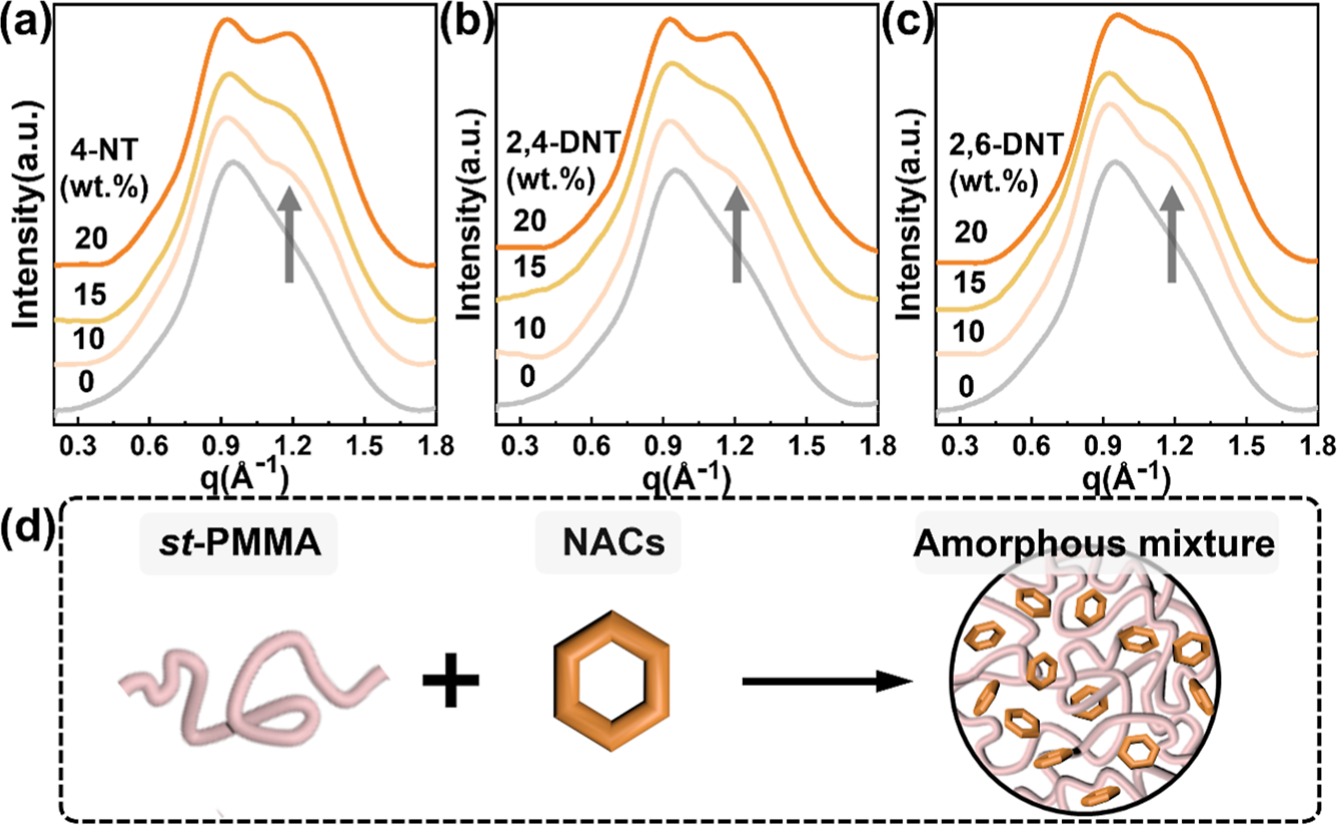
Composition-dependent WAXD profiles of the *st*-PMMA/NAC mixtures, including (a) *st*-PMMA/4-NT, (b) *st*-PMMA/2,4-DNT and (c) *st*-PMMA/2,6-DNT. (d) Schematic illustration of the blending behavior in the *st*-PMMA/NACs mixtures.

### Molecular Carrier-Induced self-Assembly in the *st*-PMMA Complex system

In biological systems, the catalytic function of protein enzymes often relies on the cooperative role of coenzymes, which modulate the conformation of the active site and enhance substrate binding.^10^ Inspired by this paradigm, we explored whether pyrene guests, an electron-rich aromatic molecule known to form CT complexes with electron-deficient NACs,^28,32^ can serve as a molecular carrier to mediate the encapsulation of NACs into the *st*-PMMA supramolecular helix. The progressive structural evolution in the carrier-mediated supramolecular encapsulation mechanism is conceptually illustrated in Figure 4a. First, as shown in Figure 4b, titrating 2,4-DNT into a *st*-PMMA/pyrene solution ([*st*-PMMA] = 2 × 10^–2^ M; pyrene E-ratio = 10 wt %) leads to a gradual color change from colorless to light yellow as the pyrene/2,4-DNT molar ratio approaches 10:10. UV–vis spectroscopy further confirms that the pristine *st*-PMMA/pyrene solution lacks absorption in the visible light range, while a broad CT absorption band between λ = 380–430 nm progressively appears upon 2,4-DNT addition, indicating the formation of CT complexes (pyrene/2,4-DNT) in the solution. FL titration spectra in Figure 4c provide additional insight into the molecular interactions between *st*-PMMA, pyrene, and 2,4-DNT. Before titration, the *st*-PMMA/pyrene dilute solution exhibits a stronger FL emission peak at approximately λ_em_ = 475 nm, characteristic of pyrene excimers, alongside a weaker peak at λ_em_ = 390 nm corresponding to pyrene monomers. These spectral features suggest that *st*-PMMA hosts bring pyrene molecules into close proximity through supramolecular wrapping, thereby promoting excimer formation in solution.^26^ Upon incremental addition of 2,4-DNT, the excimer FL at 475 nm is significantly quenched, as shown in Figure 4c. This quenching behavior arises from the formation of a nonradiative CT complex of pyrene (π-electron donor) and 2,4-DNT (π-electron acceptor), which disrupts the excimer configuration and diminishes its characteristic emission. Similar spectral behaviors were also observed with other NACs (e.g., 4-NT, 2,6-DNT), as shown in Figures S5 and S6. To further substantiate the formation of CT complexes in the solution state, we also conducted ^1^H NMR titration experiments in THF-*d*_8_ using *st*-PMMA/pyrene/2,4-DNT system (Figure 4d). The spectrum of the *st*-PMMA/pyrene/2,4-DNT solution exhibited marked deviations from those of the binary counterparts, clearly indicating a fast-exchange regime on the NMR time scale as a result of dynamic CT complexation.^33^ Upon adding 2,4-DNT, all the H signals of pyrene shifted upfield, reflecting the CT interaction from the electron-deficient NACs.^34^ Simultaneously, the H signals of 2,4-DNT also undergo upfield shifts, attributed to shielding effects induced by electron-rich pyrene. These chemical shift changes provide compelling evidence that CT interaction between pyrene and 2,4-DNT brings out the pyrene-tagged NACs complexes spontaneously in the solution phase, prior to solid-state assembly, as schematically depicted in Figure 4a. The comparable complexation-induced shifts of proton signals were also observed in the NMR spectra of *st*-PMMA/pyrene mixtures with other NACs like 4-NT and 2,6-DNT (Figures S5 and S6).

**Figure 4:**
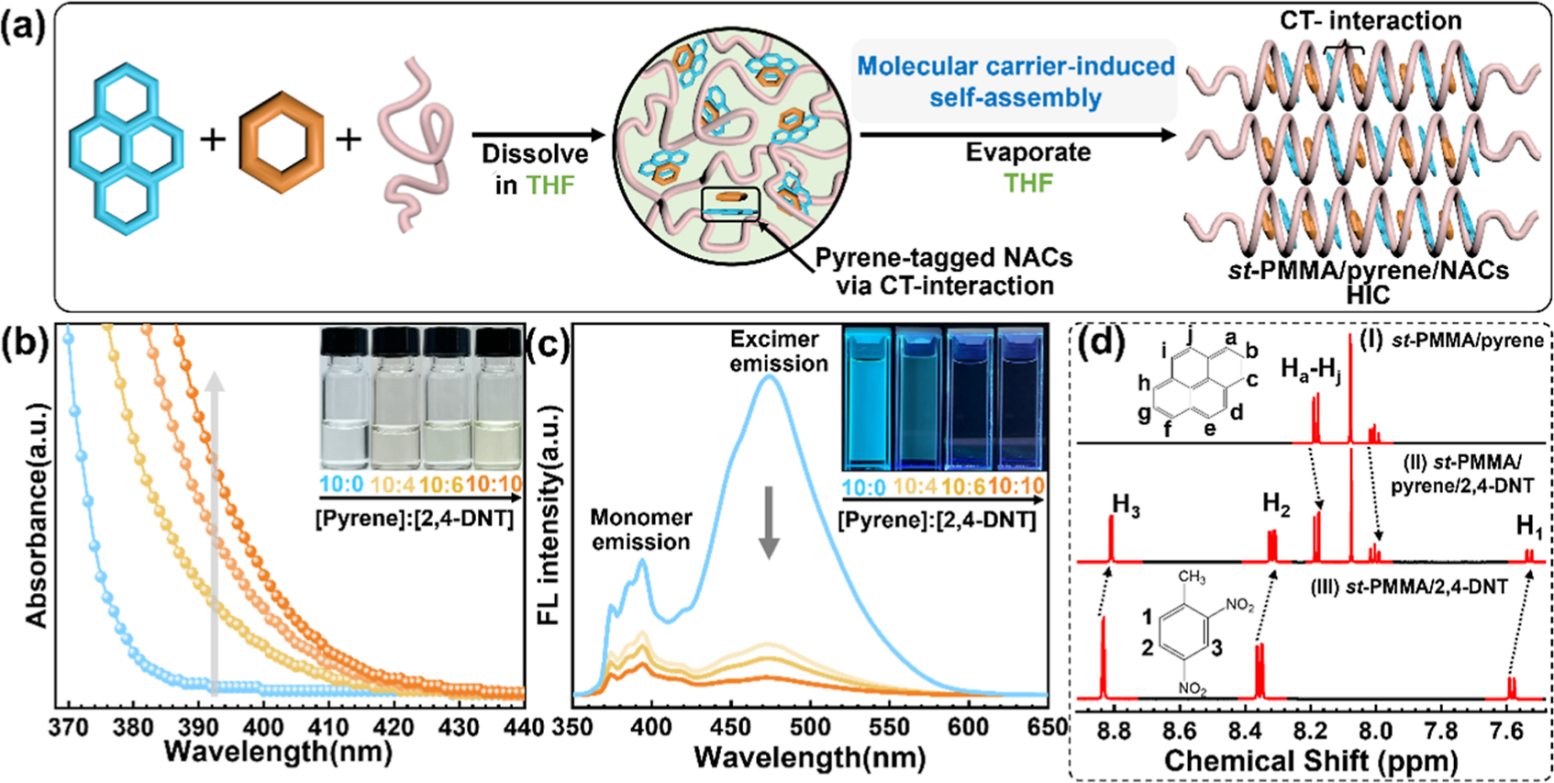
(a) Schematic illustration of molecular carrier-induced self-assembly in the *st*-PMMA/pyrene/NAC complex system. (b) UV–vis spectrum of the THF solutions of *st*-PMMA/pyrene, and *st*-PMMA/pyrene/2,4-DNT mixtures with various molar ratio of pyrene/2,4-DNT. Pyrene content relative to *st*-PMMA is 10 wt %. Insets are the corresponding photographs. (c) FL spectra (λ_ex_ = 340 nm) of the THF solutions of *st*-PMMA/pyrene, and *st*-PMMA/pyrene/2,4-DNT mixtures with various molar ratio of pyrene/2,4-DNT. Insets are the corresponding photographs excited by UV lamp (λ_ex_ = 254 nm). (d) Partial ^1^H NMR spectrum (298 K, THF-*d*_8_) of (I) *st*-PMMA/pyrene solution, (II) *st*-PMMA/pyrene/2,4-DNT solution with pyrene/2,4-DNT molar ratio = 10:10, and (III) *st*-PMMA/2,4-DNT solution. The *st*-PMMA concentration is 2 mM.

The CT complexation between pyrene and NACs in solution produces pyrene-tagged NACs, which are then coencapsulated into the *st*-PMMA supramolecular helix during the transition to the solid state, as illustrated in Figure 4a. Casting the *st*-PMMA/pyrene/2,4-DNT solution into films results in a transparent morphology that gradually turns yellow with increasing 2,4-DNT content (Figure 5a), mirroring the CT complex behavior in the solution state. The persistent FL quenching at higher pyrene/2,4-DNT molar ratios (Figure 5b) also indicates that the CT complex appears in the *st*-PMMA matrix. To probe the structural evolution triggered by molecular carrier-induced inclusion, the synchrotron-based WAXD was employed. The 1D-WAXD profiles (Figure 5c) show that as the pyrene/2,4-DNT molar ratio approaches 10:10 in the *st*-PMMA matrix, the diffraction peaks from the *st*-PMMA helical complex become both more intense and sharper, suggesting the growth in both composition and domain size of the *st*-PMMA HICs within the semicrystalline morphology. Quantitative deconvolution of the scattering intensity areas (detailed in the Supporting Information) allows separation of contributions from the crystalline *st*-PMMA HICs and the unwrapped amorphous *st*-PMMA domains. Figure 5d shows that the crystallinity (*X*_c_) of the *st*-PMMA ternary system increases from 45% to 70% as the pyrene/2,4-DNT molar ratio reaches 10:10. Moreover, using the Scherrer equation (τ = 2π*K*/Δ*q*, with *K* = 1), we further quantified the correlation length at *q*_ptich_ to obtain the helical wrapping length (τ_ptich_). Figure 5c,d shows that the wrapping length of *st*-PMMA extends significantly from τ_ptich_ = 50 Å in the *st*-PMMA/pyrene complex to 70 Å upon formation of the *st*-PMMA/pyrene/2,4-DNT ternary HICs with the molar ratio of 10:10. In addition, *q*_helix–helix_ slightly shifts toward lower scattering angles, corresponding to an expansion in helical spacing from 17.6 Å (*q* = 0.356 Å^–1^) to 18.5 Å (*q* = 0.340 Å^–1^). These structural features provide compelling evidence for the successful encapsulation of pyrene/NACs CT complexes within the *st*-PMMA helical structures. As a result, these findings demonstrate that the pyrene guest can function as a molecular carrier, facilitating the CT-driven incorporation of NACs into the *st*-PMMA supramolecular helix, as depicted in Figure 5e.

**Figure 5:**
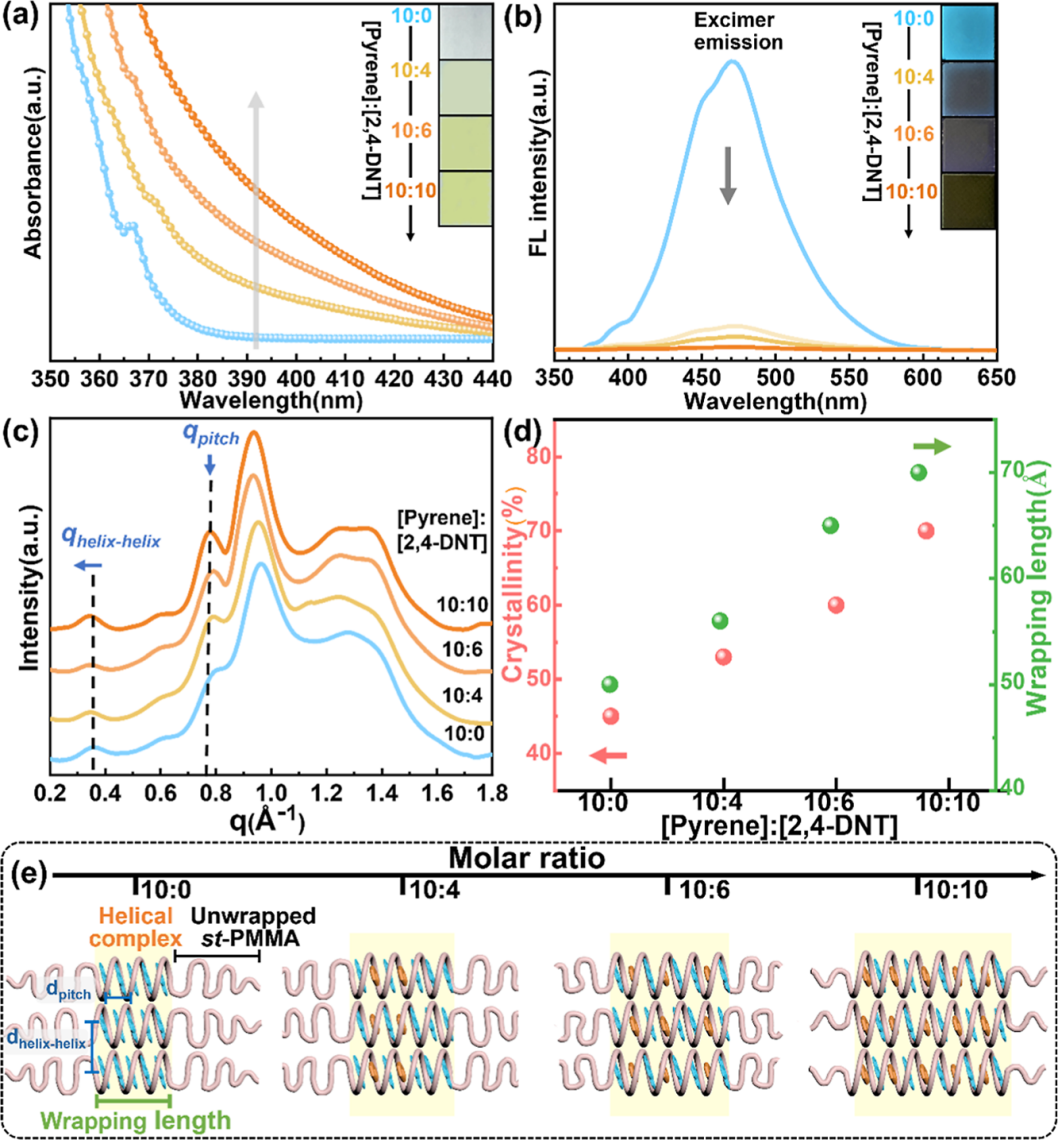
(a) UV–vis spectrum of *st*-PMMA/pyrene/2,4-DNT complex films with various molar ratio of pyrene/2,4-DNT. Insets are the corresponding photographs. (b) Corresponding FL spectra (λ_ex_ = 340 nm) of *st*-PMMA/pyrene/2,4-DNT complex films. Insets are the corresponding photographs excited by a UV lamp (λ_ex_ = 254 nm). (c) WAXD profiles of the *st*-PMMA/pyrene/2,4-DNT complex films. (d) Composition (*X*_c_) and wrapping length (τ_pitch_) of *st*-PMMA/pyrene/2,4-DNT HICs in the complex films with varying molar ratios of pyrene/2,4-DNT. (e) Schematic illustration of the self-assembled structure of the *st*-PMMA/pyrene/2,4-DNT complex with varying molar ratios of pyrene/2,4-DNT. Note: The E-ratio of pyrene in the *st*-PMMA HICs is fixed as 10 wt %.

### Detection of NAC Compounds in the Aqueous Medium

The detection of nitroaromatic compounds has garnered significant attention due to their potential safety hazards and widespread use in explosives and environmental contaminants.^35,36^ To evaluate the sensing capability of the *st*-PMMA/pyrene supramolecular complex, we prepared the transparent thin films with pyrene E-ratio = 10 wt % via the drop-casting method. We soaked them to dilute aqueous solutions of NACs, such as 4-NT, 2,4-DNT, and 2,6-DNT compounds, as shown in Figure 6a. The photos in Figure 6b record the FL quenching of the *st*-PMMA/pyrene transparent film to the 2,4-DNT_(aq)_ with 2:8 (v/v) of acetone/H_2_O solvent mixture under a low [2,4-DNT] range of 100 to 10 ppm. This FL-quenching observation suggests that NAC molecules in aqueous solution can readily diffuse into the amorphous domains of the transparent st-PMMA/pyrene film. Subsequently, facilitated by the dynamic nature of carrier-assisted self-assembly, NAC molecules intercalate into the *st*-PMMA supramolecular helices via charge-transfer (CT) interactions, thereby disrupting excimer formation and leading to pronounced FL quenching. Moreover, Figure 7 further displays the FL-quenched response times of the *st*-PMMA/pyrene complex films upon different NAC concentrations in the range of 100 to 10 ppm. At [NAC] = 100 ppm, the *st*-PMMA/pyrene complex films exhibited the faster response time with dinitrotoluenes (*t*_quench_ = ca. 5 min) than 4-NT (*t*_quench_ = ca. 12 min) since the pyrene has the stronger CT interaction with the 2,4-DNT and 2,6-DNT, as evidenced in spectroscopic analysis (Figures 4, S5 and S6).

**Figure 6:**
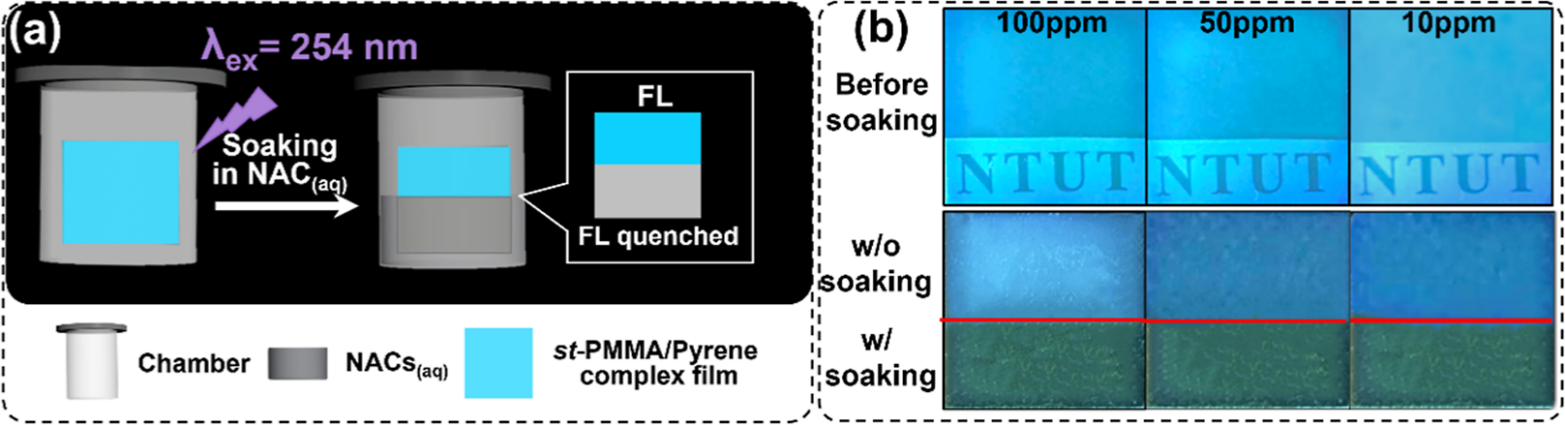
(a) Schematic of chromic response evaluation of *st*-PMMA/pyrene complex films in dilute aqueous solutions of NACs compounds, where the v/v of acetone/H_2_O is 2:8. (b) FL images of the transparent *st*-PMMA/pyrene complex films with E-ratio of pyrene = 10 wt % before/after immersion into NAC aqueous solution with concentration of 100, 50, and 10 ppm.

**Figure 7:**
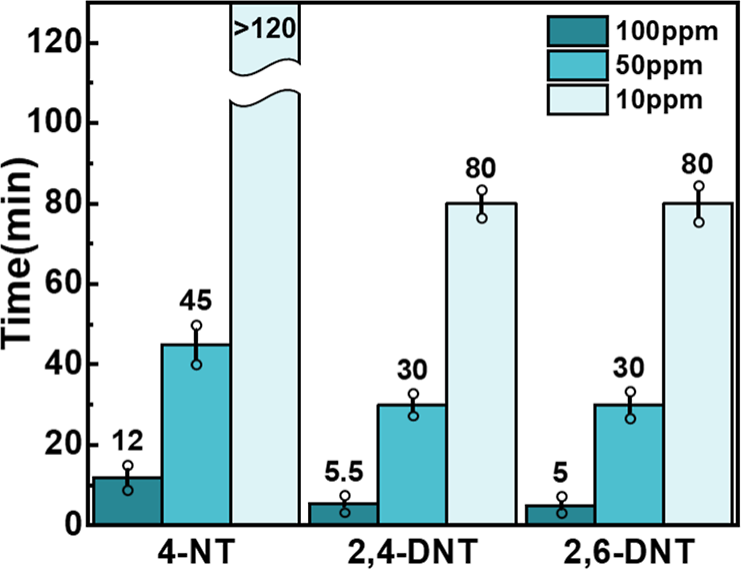
FL-quenched response time to the NACs in the aqueous solution. Data are averaged from five independent samples (*n* = 5).

Additionally, to assess the advantage of the carrier-induced self-assembled strategy, a comparative experiment was conducted using *it*-PMMA/pyrene blend films. As shown in Figure S10, the *it*-PMMA/pyrene blend film also exhibits FL quenching upon exposure to NACs_(aq)_, whereas it appears opaque, compared to the transparent *st*-PMMA/pyrene complex films. Structural analysis in Figure S11 confirms that the opacity of the *it*-PMMA/pyrene film originates from the formation of pyrene crystals since *it*-PMMA fails to wrap pyrene molecules. In contrast, the *st*-PMMA/pyrene complex exhibits a uniform and transparent morphology before/after the NAC detection. To the best of our knowledge, most transparent and colorimetric sensing materials rely on synthetic methods to tailor the stimuli-responsive polymer structures.^37,38^ Our results demonstrate that supramolecular strategies, based on molecular carrier-mediated inclusion, can afford transparent, structurally uniform films for the NAC sensing, highlighting a facile route toward the novel stimuli-responsive soft materials.

## Conclusions

This study unveils a bioinspired supramolecular strategy that employs pyrene guests as the molecular carrier to facilitate the encapsulation of nonbinding, electron-deficient NACs into the helical cavity of *st*-PMMA hosts. Structural characterization confirms that pyrene guests can be encapsulated into the *st*-PMMA hosts (*rr* = 86%) at concentrations up to 20 wt %, forming the HICs. In contrast, due to their weak binding affinity with *st*-PMMA, NACs cannot induce supramolecular assembly, resulting in an amorphous mixture. However, in the *st*-PMMA/pyrene/NACs ternary system, CT interactions serve as the driving force, first enabling the formation of pyrene/NAC complexes in solution. These pyrene-tagged NAC complexes are subsequently coencapsulated into the *st*-PMMA helical hosts during the transition to the solid state. Spectroscopic and structural characterization confirms that the CT complexation within the helical cavity of *st*-PMMAs effectively disrupts pyrene excimer formation, resulting in rapid fluorescence quenching. Moreover, the transparent *st*-PMMA/pyrene films exhibit pronounced stimuli-responsive fluorescence behavior, enabling efficient detection of explosive NACs in aqueous environments. As a result, this study establishes a generalizable carrier-induced coassembly strategy that significantly broadens the functional scope of helical polymers, with implications for future applications in chemical sensing, nanoconfined catalysis, and selective guest encapsulation in smart materials.

## Experimental Section

### Materials

*st*-PMMA (*M*_w_ = 35 kg/mol and rr content = 86%) was purchased from Polymer Source Inc. All other reagents, such as pyrene, 4-nitrotoluene (4-NT), 2,4-dinitrotoluene (2,4-DNT), 2,6-dinitrotoluene (2,6-DNT), and solvents, were purchased from Sigma-Aldrich and were used without purification. *it*-PMMA was purchased from Scientific Polymer Products Inc. The *rr* content of the *st*-PMMA was measured using ^1^H nuclear magnetic resonance (NMR) spectrometry. NMR spectra were recorded using an Agilent Unity-400 NMR spectrometer, where CDCl_3_ was employed as a deuterated solvent to identify the molecular structures at 25 °C. The number-average molecular weight (*M*_n_) and polydispersity index (PDI) of the *st*-PMMA were determined using a gel permeation chromatograph (GPC) equipped with a JASCO liquid chromatograph, comprising a JASCO PU-4180 pump, JASCO RI-4030 detector, and Stragel columns (HR1, HR2, and HR4). Tetrahydrofuran (THF) was utilized as the eluent at a flow rate of 1.0 mL min^–1^ and temperature of 30 °C. The measurements were performed at 30 °C.

### Preparation of *st*-PMMA Complex Films

Thin films of *st*-PMMA/pyrene, *st*-PMMA/NACs, and *st*-PMMA/pyrene/NACs were fabricated using the *st*-PMMA (*M*_w_ = 35 kg/mol; *rr* = 86%). Initially, 20 mg of *st*-PMMA was fully dissolved in 0.5 mL of THF under continuous stirring for 2 h to obtain the concentration of *st*-PMMA with 10^–2^ M. Pyrene and/or nitroaromatic compounds (NACs) were then added to the solution, followed by additional stirring until a homogeneous mixture was obtained. The weight content of pyrene or NACs guests relative to the *st*-PMMA host was systematically varied from 0 to 20 wt %. For the preparation of ternary *st*-PMMA/pyrene/NACs complex systems, NACs were introduced into the *st*-PMMA/pyrene solution at predetermined molar ratios to pyrene (4:10, 6:10, and 10:10 by molar quantity). Subsequently, 150 μL of each solution was drop-cast onto clean glass substrates and allowed to dry, yielding uniform and transparent thin films.

### Differential Scanning Calorimetry

Differential scanning calorimetry (DSC) measurements were performed using a TA Instruments Discovery Series 2500, equipped with an RSC 90 cooling system. Nitrogen was used as the purge gas, and the samples were scanned from 40 to 180 °C at a scan rate of 10 °C min^–1^.

### Ultraviolet–Visible Spectrometer

UV–vis absorption spectra were recorded using an Agilent Cary 5000 UV-NIR spectrometer in the transmission geometry at normal incidence within the wavelength range λ of 200–700 nm. For the *st*-PMMA/pyrene solution measurement, the *st*-PMMA was first dissolved in THF with a concentration of 2.5 × 10^–2^ M. Later, the pyrene powders were added to the *st*-PMMA solution with a mixing ratio of 10 wt % with respect to the *st*-PMMA. As for preparing the *st*-PMMA/pyrene/NACs solution, the NACs with a molar ratio to pyrene (4:10, 6:10, and 10:10 by molar quantity) were added to the *st*-PMMA/pyrene solution. The spectra of these dilute solutions were collected in a 2 mm quartz cuvette. Moreover, thin-film samples were prepared using the drop-casting method onto quartz substrates for UV–vis measurements.

### Fluorescence Spectroscopy

Fluorescence emission spectra were acquired using a fluorescence spectrophotometer (JASCO FP-8500). The spectra of dilute solutions were collected in a 2 mm quartz cuvette, with the same sample concentration used in the UV–vis analysis. Moreover, thin-film samples were prepared using the drop-casting method with 100 μL of the THF solution (2.5 × 10^–2^ M) onto quartz substrates for the thin-film fluorescence measurements.

### Optical Microscopy

Optical microscopy (OM) images of the *st*-PMMA complex films and pyrene crystalline morphology were recorded using a Leica DM2700 optical microscope.

### Wide-Angle X-ray Diffraction

Wide-angle X-ray diffraction (WAXD) measurements were performed on *st*-PMMA/pyrene, *st*-PMMA/NACs, and *st*-PMMA/pyrene/NACs complex films at the TLS-BL13A1 and TPS-BL13A beamlines of the National Synchrotron Radiation Research Center (NSRRC), Taiwan. Diffraction patterns were collected using MarCCD and Eiger X 1 M detectors with X-ray wavelengths of 1.0274 Å and 0.827 Å, respectively. The sample-to-detector distances were set at 201.43 mm and 328.45 mm. The scattering vector *q* was determined using the equation *q* = 4πsin­(θ)/λ, where θ is the scattering angle and λ is the X-ray wavelength.

## Supplementary Material



## Notes

Deconvolution description of WAXD data; ^1^H NMR and GPC spectra of *st*-PMMA; OM images of *st*-PMMA/NACs films; Deconvoluted WAXD profiles of *st*-PMMA/NACs blend films; WAXD profiles of pyrene crystals and *it*-PMMA/pyrene blend film; UV–visible spectrum, FL spectrum, and ^1^H NMR for the *st*-PMMA/pyrene/NACs solution and complex films; OM images of *st*-PMMA/pyrene and *it*-PMMA/pyrene films before/after immersion into NAC aqueous solution (PDF)
