# Supplementary material for: Molecular Carrier-Assisted Self-Assembly of st-PMMA Helical Complexes for Fluorescence Sensing of Nitroaromatics in Aqueous Medium
Source: ACS Appl Mater Interfaces. 2025 Sep 11;17(38):53942–50. doi: 10.1021/acsami.5c13301 (PMC12464911; doi:10.1021/acsami.5c13301)
Supplement: Supplementary file 1 [file am5c13301_si_001.pdf]

Supporting Information

Molecular Carrier-Assisted Self-Assembly of *st*-PMMA Helical Complexes for Fluorescence Sensing of Nitroaromatics in Aqueous Medium

*Yu-Hao Wang, Wen-Tsung Tseng, and Kuan-Yi Wu**

Department of Chemical Engineering and Biotechnology, National Taipei University of Technology, Taipei 10608, Taiwan

Corresponding Authors

E-mail: kywu@ntut.edu.tw

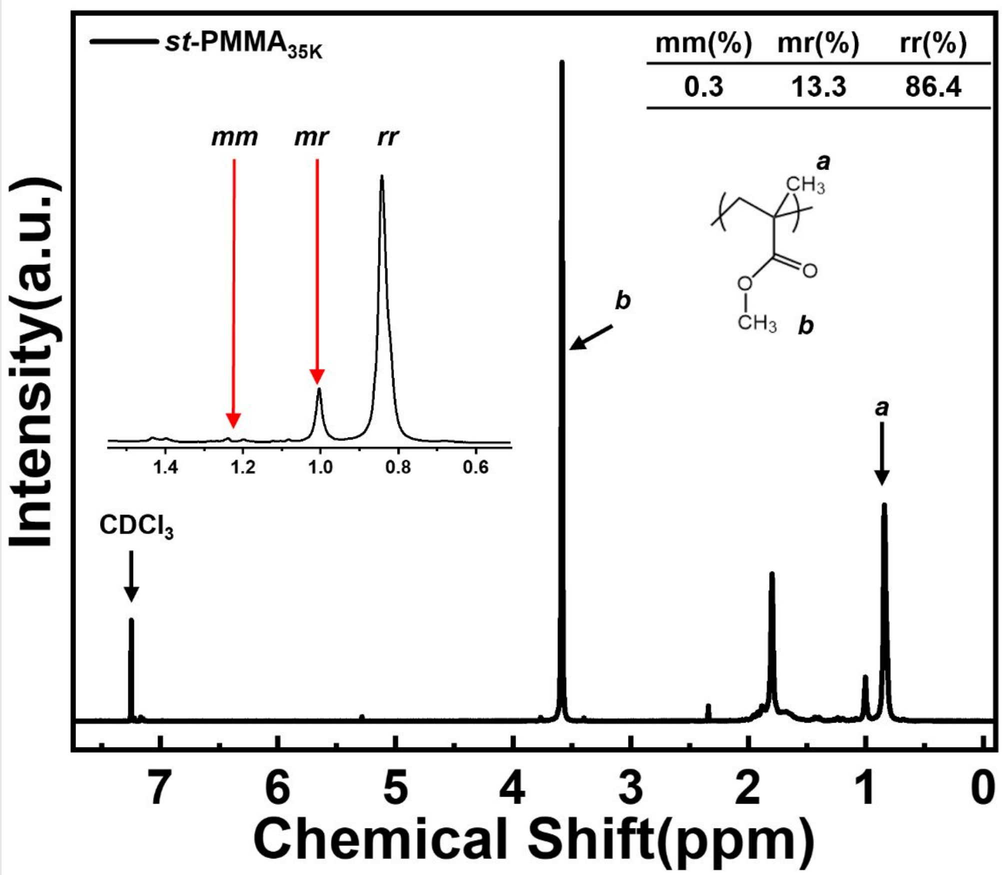

Figure S1. ^{1}H NMR spectra of *st*-PMMA with *rr* content of 86%. The spectra were measured in $CDCl_3$ at 25 °C.

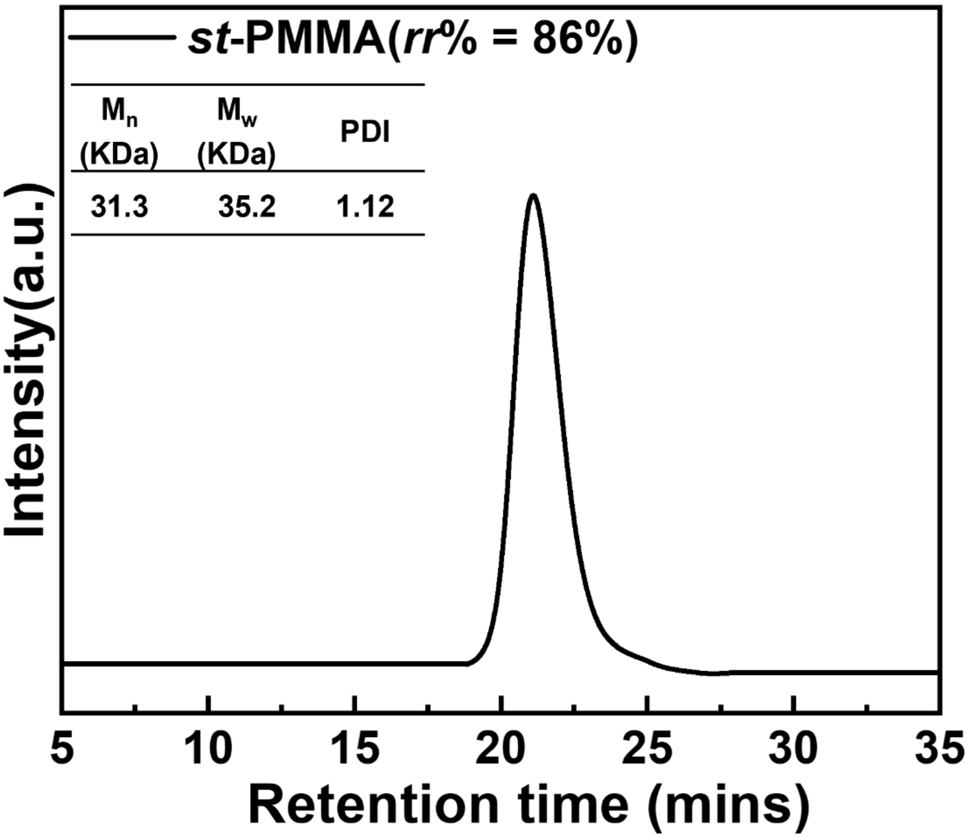

Figure S2. The gel permeation chromatography (GPC) analysis of *st*-PMMA with *rr* content = 86 %.

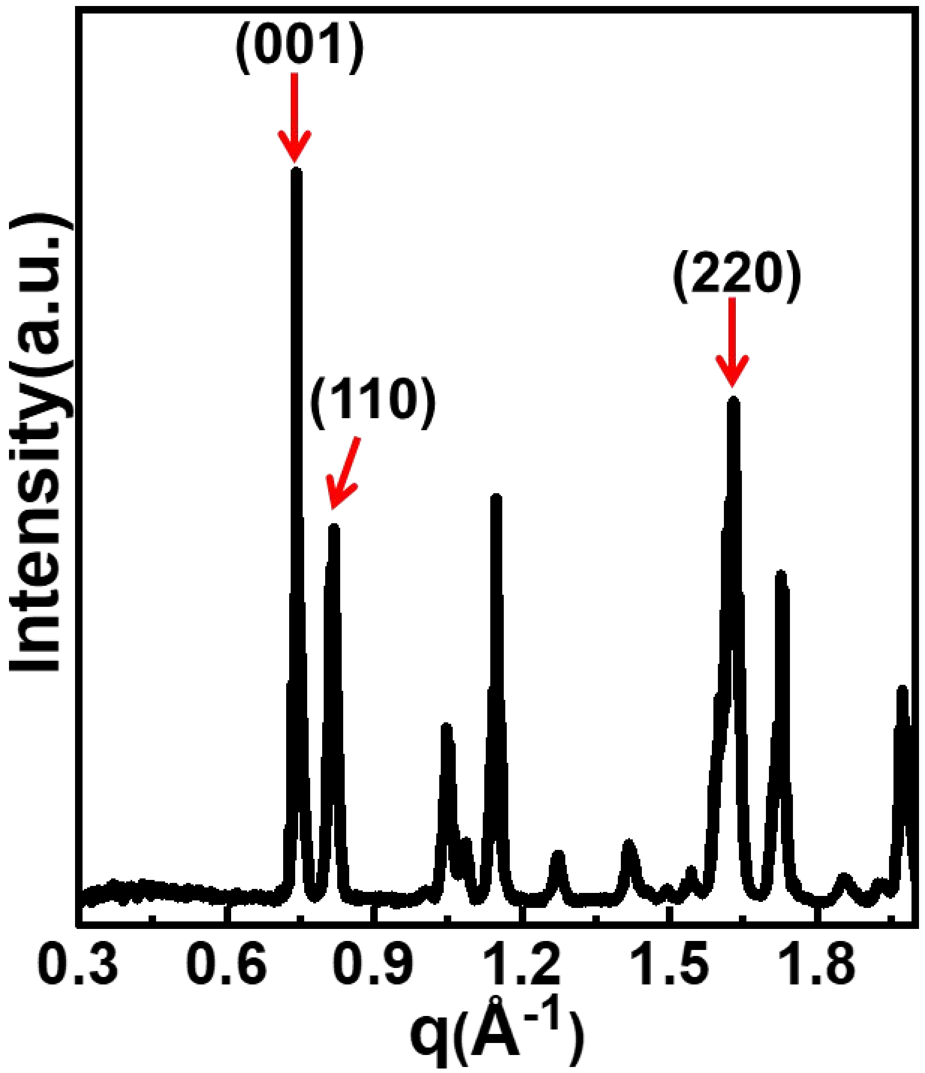

Figure S3. WAXD profile of pure pyrene crystals.

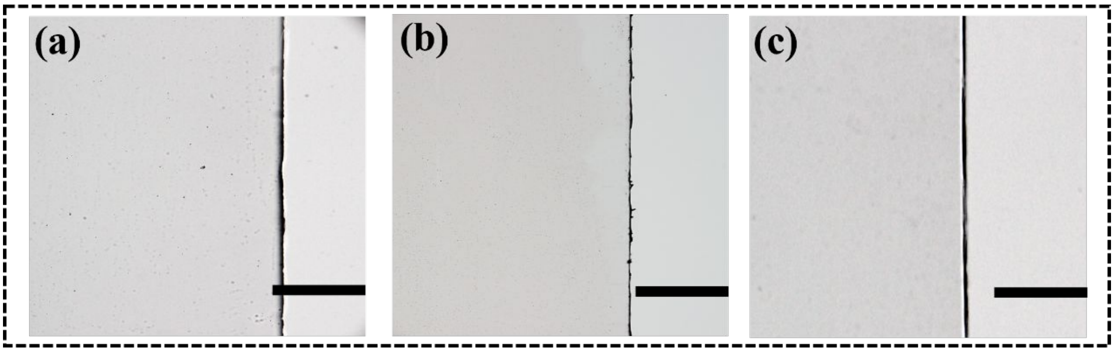

Figure S4. OM graphs of the homogenous *st*-PMMA/NACs blending films, including (a) *st*-PMMA/4-NT, (b) *st*-PMMA/2,4-DNT and (c) *st*-PMMA/2,6-DNT. Note: concentration of NACs in the *st*-PMMA mixture is 20 wt.%. (scale bar：100 μm)

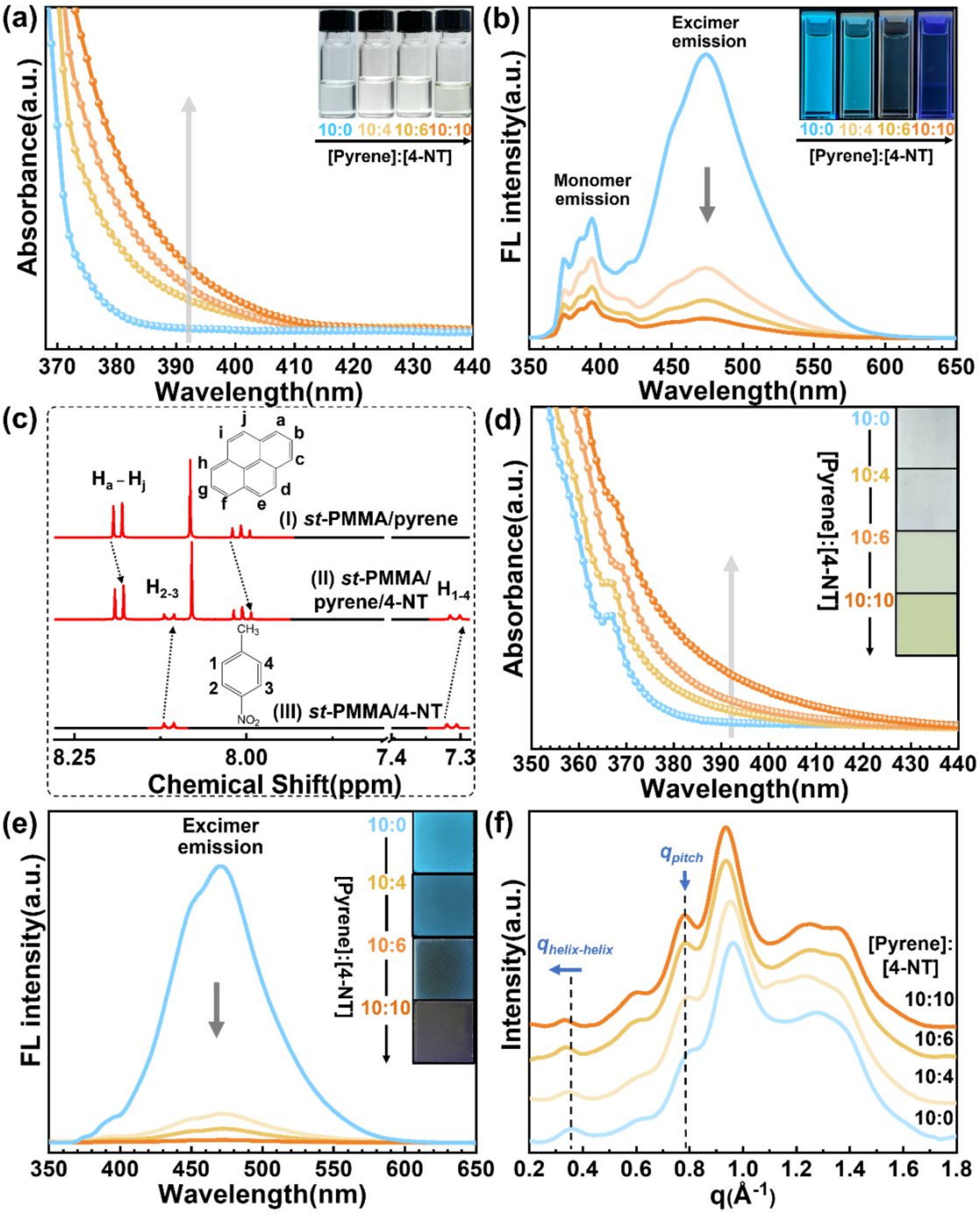

Figure S5. Structural characterization of the *st*-PMMA/pyrene/4-NT system (a) UV-VIS spectrum of the THF solutions of *st*-PMMA/pyrene and *st*-PMMA/pyrene/4-NT. Insets are the corresponding photographs. Note: pyrene concentration in the *st*-PMMA/THF solution is 10 wt.%. (b) FL spectra of the THF solutions of *st*-PMMA/pyrene and *st*-PMMA/pyrene/4-NT. Insets are the corresponding photographs excited by a UV lamp (λ_{ex} = 254 nm). (c) Partial ^{1}H NMR spectrum (298 K, THF-d_8) of (I) *st*-PMMA/pyrene solution, (II) *st*-PMMA/pyrene/4-NT solution with a molar ratio of 4-NT = 10:10, and (III) *st*-PMMA/4-NT solution. The *st*-PMMA concentration is 2 mM. (d) UV-VIS spectrum of *st*-PMMA/pyrene/4-NT complex films. (e) FL spectrum of *st*-PMMA/pyrene/4-NT complex films. (f) WAXD profiles of the *st*-PMMA/pyrene/4-NT complex films with various molar ratio.

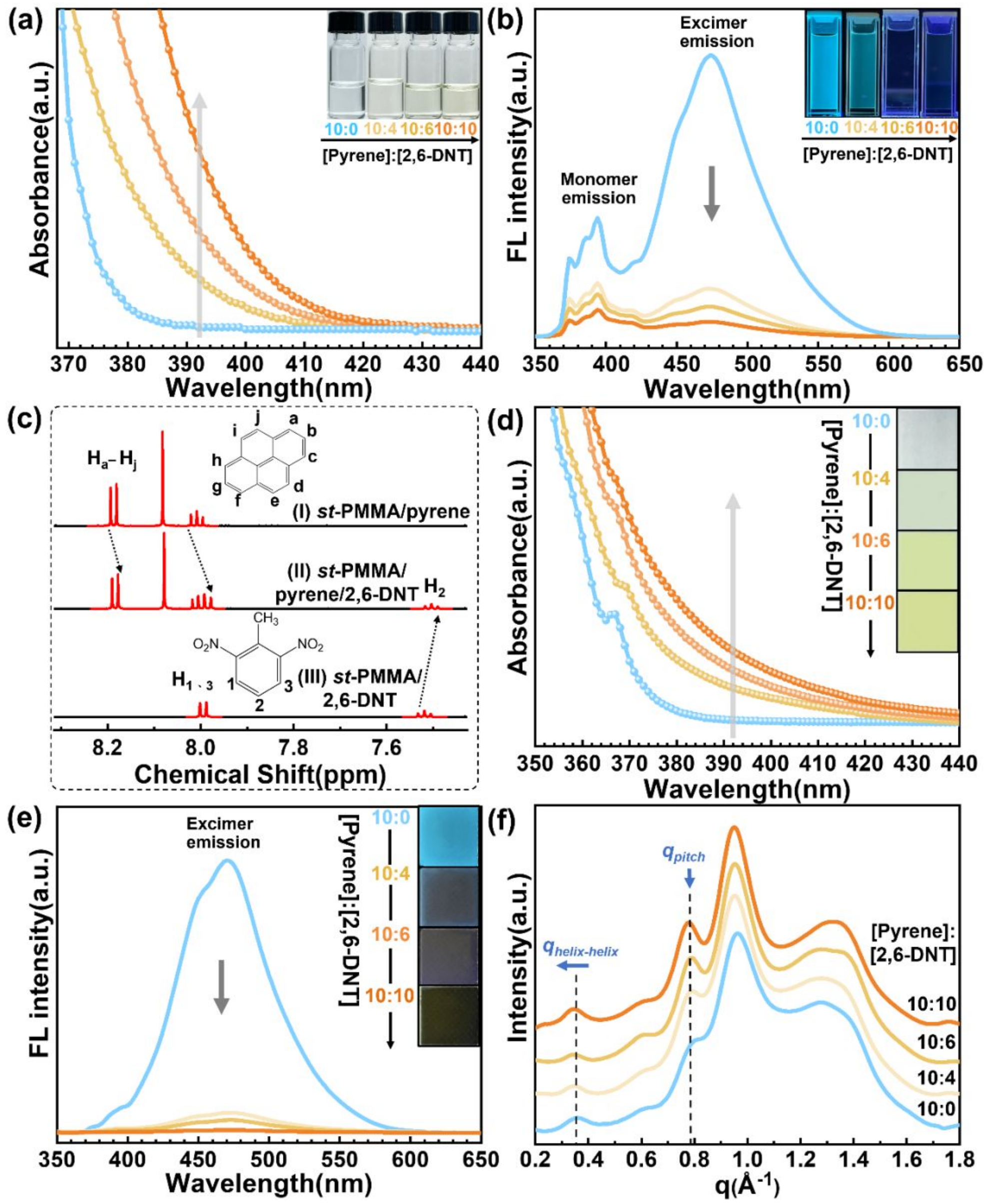

Figure S6. Structural characterization of the *st*-PMMA/pyrene/2,6-DNT system (a) UV-VIS spectrum of the THF solutions of *st*-PMMA/pyrene and *st*-PMMA/pyrene/2,6-DNT. Insets are the corresponding photographs. Note: pyrene concentration in the *st*-PMMA/THF solution is 10 wt.%. (b) FL spectra of the THF solutions of *st*-PMMA/pyrene and st-PMMA/pyrene/2,6-DNT. Insets are the corresponding photographs excited by a UV lamp (λ_{ex} = 254 nm). (c) Partial ^{1}H NMR spectrum (298 K, THF-d_8) of (I) *st*-PMMA/pyrene solution, (II) the *st*-PMMA/pyrene/2,6-DNT solution with molar ratio = 10:10, and (III) *st*-PMMA/2,6-DNT solution. The *st*-PMMA concentration is 2 mM. (d) UV-VIS spectrum of *st*-PMMA/pyrene/2.6-DNT complex films. (e) FL spectrum of *st*-PMMA/pyrene/2,6-DNT complex films. (f) WAXD profiles of the *st*-PMMA/pyrene/2,6-DNT complex films with various molar ratio.

Composition evolution of the *st*-PMMA/pyrene/2,4-DNT helical bundles and amorphous *st*-PMMA domain in the *st*-PMMA complex morphology

The *st*-PMMA is an inherently amorphous polymer; however, its co-assembly with pyrene and NACs enables the formation of the *st*-PMMA helical inclusion complex. On a larger scale, this co-assembly leads to the emergence of a two-phase morphology in the *st*-PMMA complex films, consisting of crystalline helical bundles of *st*-PMMA complex embedded within an amorphous matrix of unwrapped *st*-PMMA chains. To elucidate the structural evolution induced by molecular encapsulation, composition-dependent wide-angle X-ray diffraction (WAXD) analysis was employed. As shown in **Figure 5c**, the appearance of sharp diffraction features begins at a pyrene loading of 10 wt.%, and the peak intensity increases significantly when the 2,4-DNT/pyrene molar ratio reaches 10:10, indicating the progressive formation of ordered helical structures. Therefore, the WAXD profiles in **Figure 5c** can be deconvoluted to the scattering contributions of the amorphous and crystalline domains, shown in **Figure S7**. Orange and blue lines represent scattering peaks from the amorphous structure of *st*-PMMA and crystalline structure of the *st*-PMMA complex, respectively. To obtain the composition of the helical bundles of *st*-PMMA helical complexes, we can use the equation: Crystallinity (X_c) = $A_{crystalline}/(A_{crystalline} + A_{amorphous})$, where the $A_{crystalline}$ and $A_{amorphous}$ are the diffraction intensity area of the crystalline domain and the area of scattering intensity of the amorphous *st*-PMMA domain, respectively. As summarized in **Table S1**, the pristine *st*-PMMA/pyrene complex (E-ratio = 10 wt.%) exhibits an X_c of approximately 45%. Upon gradually increasing the 2,4-DNT/pyrene molar ratio to 10:10, the X_c increases to ~70%, supporting that pyrene acts as a molecular carrier that enables efficient NACs incorporation into the helical domains. In addition to increased crystallinity, the encapsulation of 2,4 DNTs also induces a notable extension in the helical wrapping length of the *st*-PMMA complex, which reflects the full width at half maximum of the q_{pitch}. Thus, **Eq S1**, the Scherrer equation ($\tau = 2\pi K/\Delta q$, where τ is the correlation length of a periodic structure, Δq is the full width at half maximum of the q_{pitch}, and the shape factor, K, is equal to 1) can be applied to calculate the correlation length of helical pitch (wrapping length ; τ_{pitch}) for the *st*-PMMA inclusion complex. **Table S1** lists that the largest τ_{pitch} value of 70 Å at the molar ratio = 10:10 corresponds to the highest wrapping length of the *st*-PMMA/pyrene/2,4-DNT supramolecular helix. Besides, the analytical results of *st*-

PMMA/pyrene complex systems with other NACs like 4-NT and 2,6-DNT are also shown in **Figure S8-9** and **Table S2-3**.

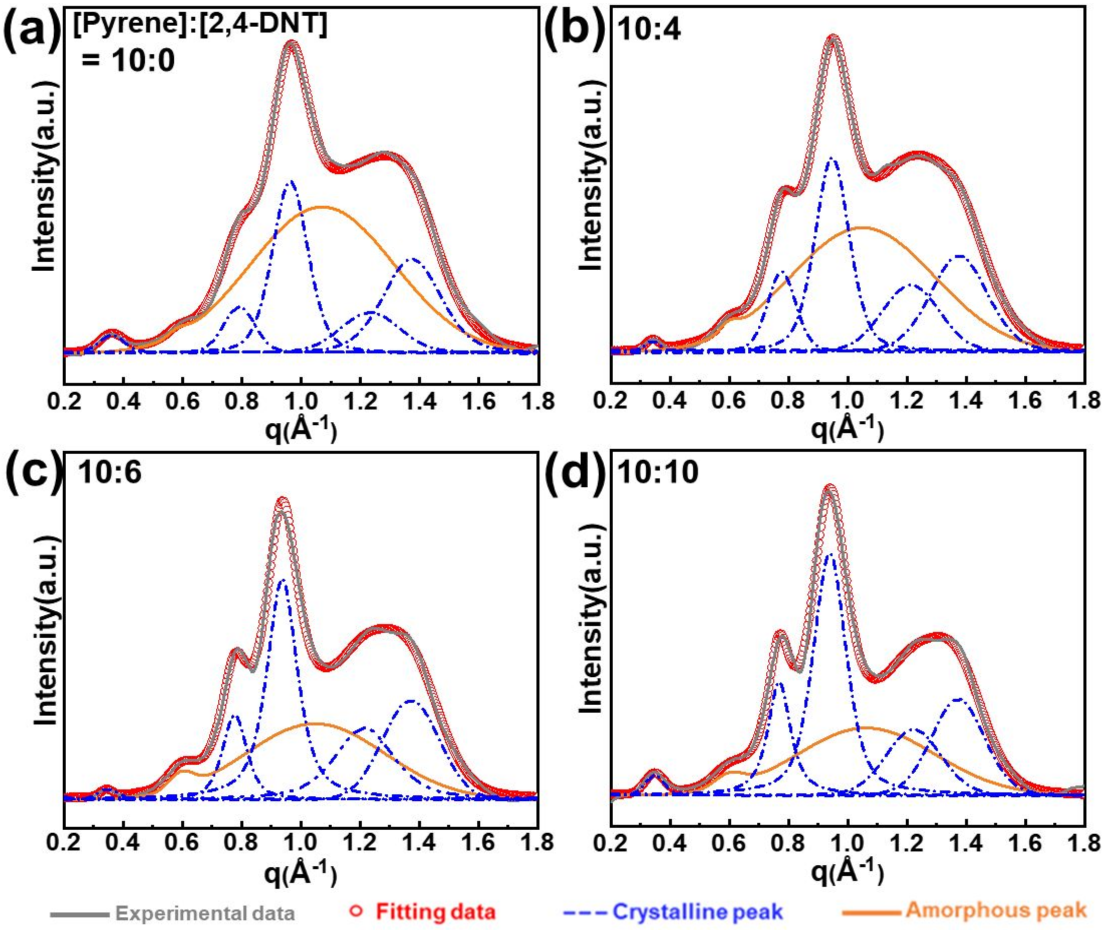

Figure S7. The WAXD profiles (grey line) and the deconvolution profiles of the *st*-PMMA/pyrene/2,4-DNT inclusion complex at molar ratio of 2,4-DNT to pyrene (a) 10:0 (b) 10:4 (c) 10:6 (d) 10:10. Note: the intensity area of deconvoluted scattering from the amorphous phase (orange line) and crystalline phase (dashed-blue line), the fitting data (red dot) and experimental data (grey line). Besides, the pyrene content in the *st*-PMMA complex system is fixed at E-ratio = 10 wt.%

Table S1. The crystallinity and wrapping length (τ_{ptich}) of the *st*-PMMA/pyrene/2,4-DNT complex system with various stoichiometric ratio.

Stoichiometric ratio	10:0	10:4	10:6	10:10
$A_{amorphous}$(%)	55	47	40	30
$A_{crystalline}$(%)	45	53	60	70
X_c (%)	45	53	60	70
τ_{ptich}(Å)	50	56	65	70

Note: pyrene content in the *st*-PMMA complex system is fixed at E-ratio = 10 wt.%

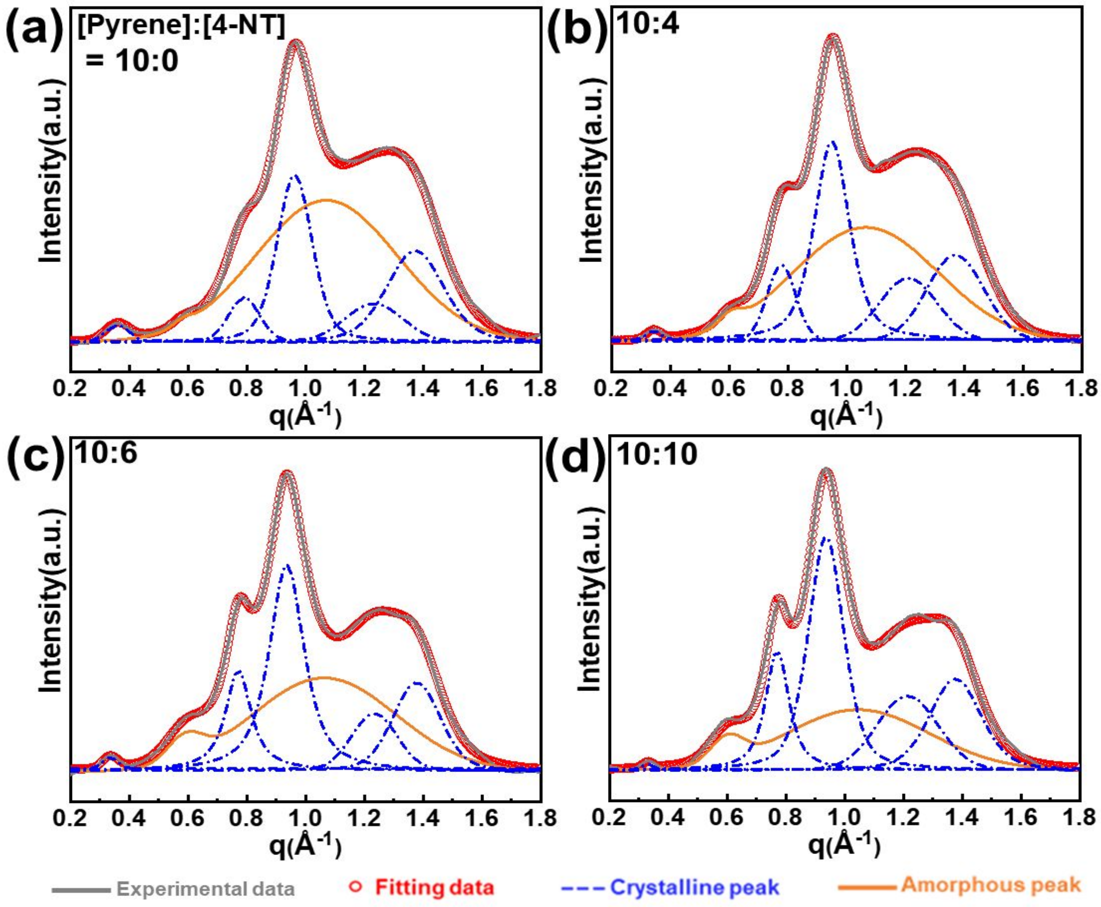

Figure S8. The WAXD profiles (grey line) and the deconvolution profiles of the *st*-PMMA/pyrene/4-NT inclusion complex at molar ratio of 4-NT to pyrene (a) 10:0 (b) 10:4 (c) 10:6 (d) 10:10. Note: the intensity area of deconvoluted scattering from the amorphous phase (orange line) and crystalline phase (dashed-blue line), the fitting data (red dot) and experimental data (grey line). Besides, the pyrene content in the *st*-PMMA complex system is fixed at E-ratio = 10 wt.%

Table S2. The crystallinity and wrapping length (τ_{ptich}) of the *st*-PMMA/pyrene/4-NT complex system with various stoichiometric ratio.

Stoichiometric ratio	10:0	10:4	10:6	10:10
$A_{amorphous}$(%)	55	46	41	29
$A_{crystalline}$(%)	45	54	59	71
X_c (%)	45	54	59	71
τ_{ptich}(Å)	50	55	61	66

Note: pyrene content in the *st*-PMMA complex system is fixed at E-ratio = 10 wt.%

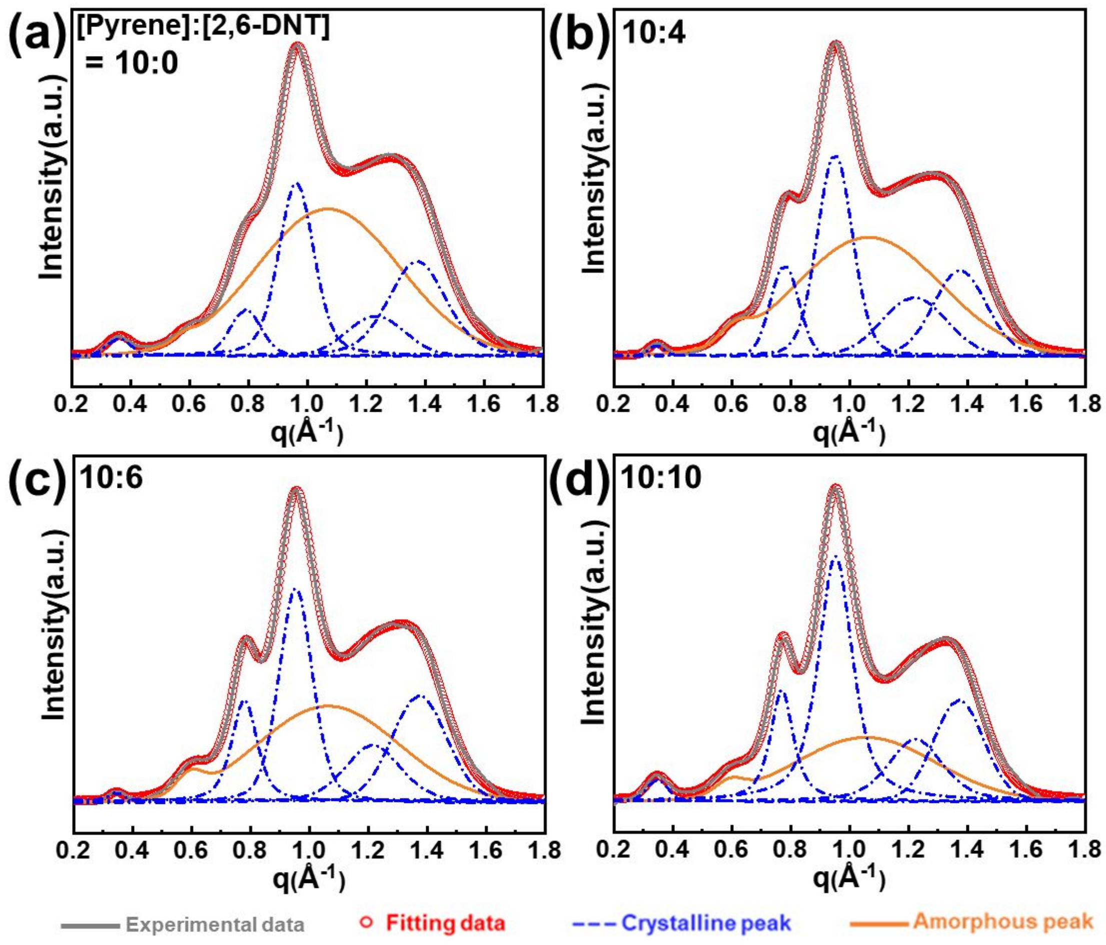

Figure S9. The WAXD profiles (grey line) and the deconvolution profiles of the *st*-PMMA/pyrene/2,6-DNT inclusion complex at molar ratio of 2,6-DNT to pyrene (a) 10:0 (b) 10:4 (c) 10:6 (d) 10:10. Note: the intensity area of deconvoluted scattering from the amorphous phase (orange line) and crystalline phase (dashed-blue line), the fitting data (red dot) and experimental data (grey line). Besides, the pyrene content in the *st*-PMMA complex system is fixed at E-ratio = 10 wt.%

Table S3. The crystallinity and wrapping length (τ_{ptich}) of the *st*-PMMA/pyrene/2,6-DNT complex system with various stoichiometric ratio.

Stoichiometric ratio	10:0	10:4	10:6	10:10
$A_{amorphous}$(%)	55	48	40	28
$A_{crystalline}$(%)	45	52	60	72
X_c (%)	45	52	60	72
τ_{ptich}(Å)	50	55	61	68

Note: pyrene content in the *st*-PMMA complex system is fixed at E-ratio = 10 wt.%

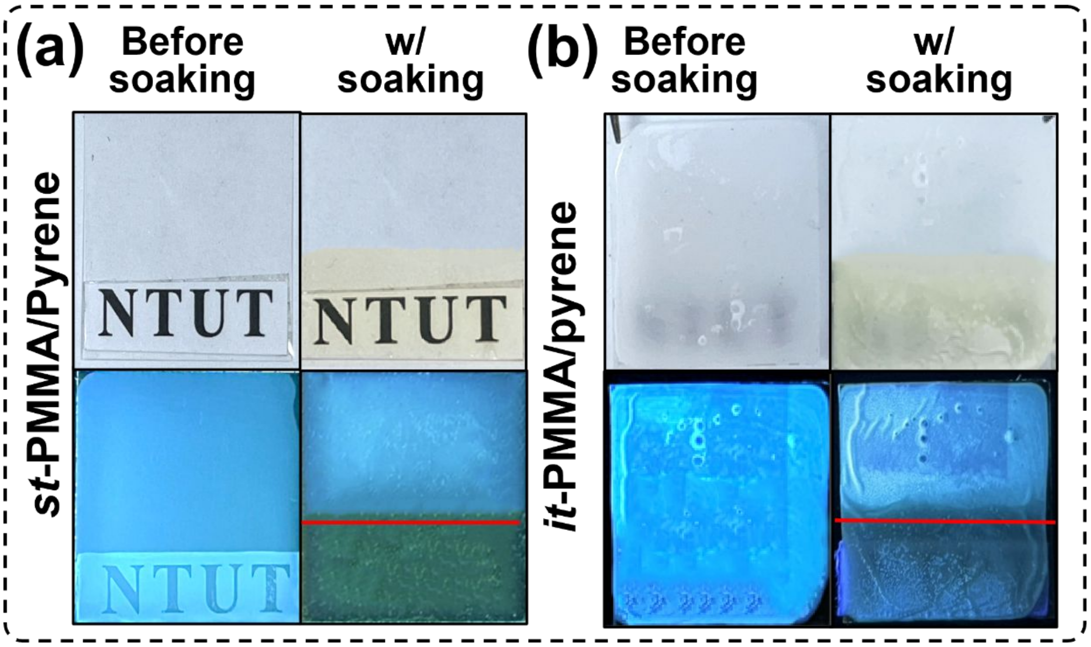

Figure S10. The opaque *it*-PMMA/pyrene blend film exhibits FL quenching upon exposure to NACs, compared to the transparent *st*-PMMA/pyrene complex films. Note: The pyrene content in these two blending films is fixed at 10 wt.%.

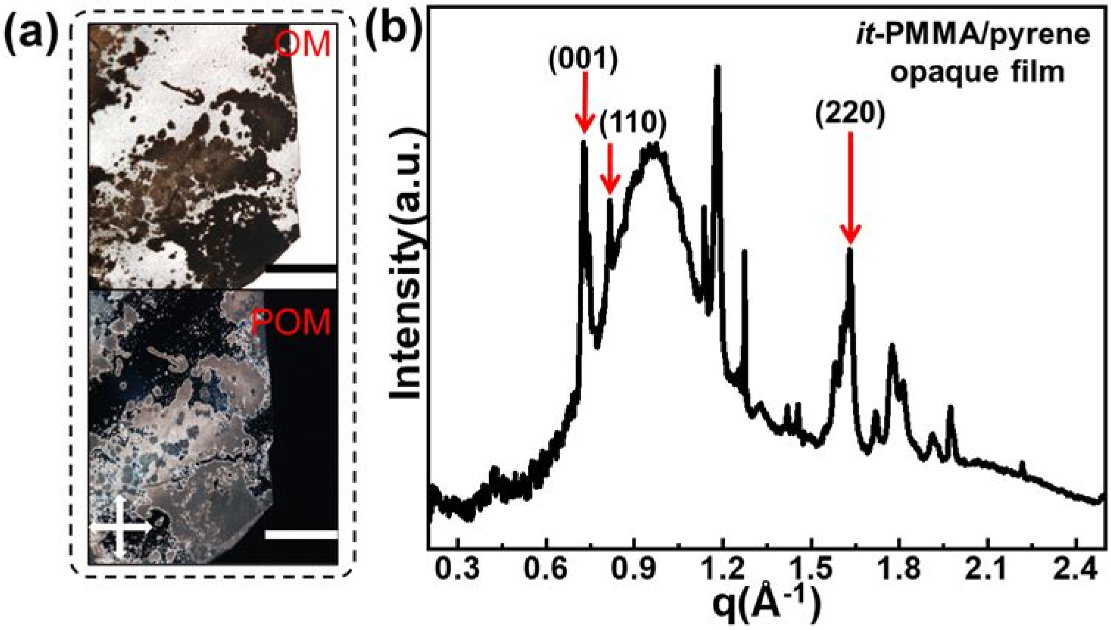

Figure S11. (a) OM and POM graphs of the opaque *it*-PMMA/pyrene film. (b) WAXD profile of opaque *it*-PMMA/pyrene blend films with [pyrene] = 10 wt.%. Note： The polarizer and the analyzer (white arrows) are in a perpendicular configuration. (scale bar：100 μm)
